# Micro-Oxygenation in Upflow Anaerobic Sludge Bed (UASB) Reactors Using a Silicon Membrane for Sulfide Oxidation

**DOI:** 10.3390/polym12091990

**Published:** 2020-09-01

**Authors:** Freddy Valdés, Priscila Rosseto Camiloti, Jan Bartacek, Álvaro Torres-Aravena, Javiera Toledo-Alarcón, Marcelo Zaiat, David Jeison

**Affiliations:** 1 Departamento de Ingeniería Química, Universidad de La Frontera. Av. Francisco Salazar, 01145 Temuco, Chile; freddyvaldesg@gmail.com; 2 Laboratory of Biological Processes, São Carlos School of Engineering, University of São Paulo (USP), 1100, João Dagnone Ave., Santa Angelina, 13.563-120 São Carlos, SP, Brazil; prcamiloti@gmail.com (P.R.C.); zaiat@sc.usp.br (M.Z.); 3 Department of Water Technology and Environmental Engineering, University of Chemistry and Technology Prague, Technicka 5, 166 28 Prague 6, Czech Republic; jan.bartacek@vscht.cz; 4 Escuela de Ingeniería Bioquímica, Facultad de Ingeniería, Pontificia Universidad Católica de Valparaíso, Av. Brasil, 2085 Valparaíso, Chile; alvaro.torres@pucv.cl (Á.T.-A.); javiera.toledo@pucv.cl (J.T.-A.)

**Keywords:** oxygen, oxidation, sulfide, membrane, UASB

## Abstract

Sulfide produced by sulphate-reducing bacteria in anaerobic reactors can seriously affect biogas quality. Microaeration has become a reliable way to remove sulfide, by promoting its oxidation. However, limited research is available regarding its application in upflow anaerobic sludge bed (UASB) reactors. In this research, silicon membranes were studied as a mechanism to dose oxygen in USAB reactors. Two configurations were tested: the membrane placed inside the reactor or in an external module. Our results show that the external membrane proved to be a more practical alternative, providing conditions for sulfide oxidation. This led to a reduction in its concentration in the liquid effluent and biogas. External membrane configuration achieved a sulfide conversion rate of 2.4 g-S m^2^ d^−1^. Since the membrane was not sulfide-selective, methane losses were observed (about 9%). In addition, excessive oxygen consumption was observed, compared to the stoichiometric requirement. As is the case for many membrane-based systems, membrane area is a key factor determining the correct operation of the system.

## 1. Introduction

High sulfate concentrations can be found in the wastewater from different industries, including pharmaceutical, food, tannery, edible oil refinery, among others [1,2,3,4]. Sulfate is generally found in nature and is chemically inert, nontoxic, and nonvolatile. However, it could affect the anaerobic digestion process during (waste) water treatment, by promoting competition between methanogens archaea and sulphate-reducing bacteria (SRB). SRB can oxidize hydrogen and various organic compounds, using sulfate as the electron acceptor, and producing hydrogen sulfide (H_2_S) [4,5,6]. H_2_S is toxic to the methane producing microbial community. Normally, maintaining a chemical oxygen demand (COD)/sulfate ratio over 10 prevents inhibiting sulfide concentrations being reached in anaerobic reactors, which, depending on the pH, can be between 50 and 800 mg L^-1^ [7,8]. High concentrations of H_2_S in the produced biogas reduces its quality, causing corrosion in the distribution lines and equipment, as well as generating undesirable odors [9].

Different strategies have been used to mitigate H_2_S production in anaerobic reactors. They include the removal of sulfate from wastewater (before feeding the anaerobic digester) by chemical precipitation [10], inhibition of the SRB using specific compounds such as molybdate [11,12], and using iron salt to oxidize the H_2_S [13]. Although these techniques are efficient in preventing or mitigating H_2_S production, they are costly and may be unsustainable.

An interesting alternative involves the microbial oxidation of H_2_S to elemental sulfur (S^0^), through the injection of microdoses of oxygen into the system [14]. Pure oxygen or air can be supplied in the recirculation line, in the liquid phase, or in the headspace of the anaerobic reactor [15]. The latter is the most used option due to its operational simplicity, since the bacterial community capable of oxidizing H_2_S can grow as a biofilm on the walls of the headspace [2,16,17]. A minimal residence time of the biogas in the headspace is decisive for achieving high removal efficiencies [14,17]. Even though several authors have reported successful results by injecting air/oxygen into the headspace of continuous stirred tank reactors (CSTRs), limited research has been reported involving upflow anaerobic sludge blanket (UASB) reactors [1,14]. It is probable that the design and the smaller headspace of UASB reactors do not provide an adequate gas residence time, which is required for effective H_2_S removal [17].

Since UASB reactors are a traditional treatment alternative, massively used worldwide for wastewater treatment, more research is needed to develop new strategies to improve H_2_S removal efficiency, such as oxygen injection into the liquid phase of the reactor. In this context, the use of membranes has been proposed as an innovative strategy to provide micro-oxygenation. This research aimed to test silicon membranes as a micro-oxygenation mechanism to promote H_2_S removal by microbial oxidation. Two configurations for micro-oxygenation were compared in a UASB reactor: immersed in the liquid phase and in an external unit connected to biogas recirculation.

## 2. Materials and Methods

### 2.1. Reactor Setup

Two reactor configurations were implemented to test the effectiveness of membranes for oxygen transfer to UASB reactors. Both configurations used chemically treated silicone tubes as membranes and involved identical UASB reactors of 2.2 L of useful volume. Chemical treatment of silicone tubes consisted of submerging them in a 70% ethanol, 30% water solution for 36 h, in order to increase the gas permeability.

**Figure 1:**
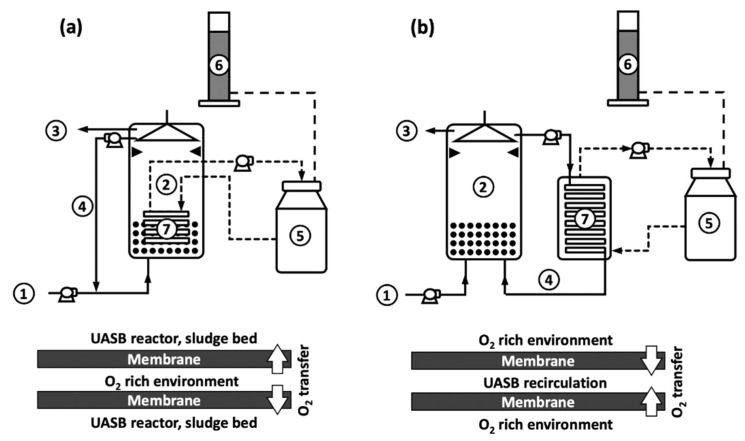
Schematic representation of reactor configurations tested in this study. (**a**) Reactor 1, with submerged silicone membrane; (**b**) Reactor 2, with external mass transfer unit. 1: Upflow anaerobic sludge bed (UASB) feed, 2: UASB reactor, 3: UASB effluent, 4: UASB recirculation, 5: oxygen container, 6: graduated cylinder, 7: silicone membrane. Scheme below each configuration depicts the direction of oxygen transfer (inside/out or outside in).

In the first configuration (Reactor 1, Figure 1a), the membrane was submerged in the sludge bed of the UASB reactor. The second configuration (Reactor 2, Figure 1b) included an external chamber that contained the membrane, through which UASB recirculation flowed. In both cases, the silicone tubes had an external diameter and thickness of 9 and 2 mm, respectively. Membranes lengths were 1.1 and 2.2 m for Reactor 1 and 2, respectively. External membrane areas were then 0.031 and 0.062 m^2^ for Reactor 1 and 2, respectively. Internal membrane areas were 0.017 and 0.035 m^2^ for Reactor 1 and 2, respectively. A shorter membrane was used in Reactor 1, since a bigger one could not be fitted inside the reactor due to space restrictions.

In both systems, pure oxygen was used to promote microaerobic conditions in the UASB reactors. During Reactor 1 operation, oxygen was circulated within the membrane using a peristaltic pump (Masterflex-Cole Parmer, Vernon Hills, Illinois, USA), at a flow of 40 mL min^-1^. Then, oxygen was transferred from the lumen of the membrane (inside/out operation for oxygen, see Figure 1a). Oxygen used for this purpose was stored in a 2 L container. In order to measure the consumed oxygen, a 1 L graduated cylinder with water was coupled to the 2 L oxygen container. Oxygen consumption in the system generated a vacuum in the 2 L container, which displaced water from the 1 L graduated cylinder to the container. Then, a decrease in water volume indicated the volume of consumed oxygen.

Gas composition in the container was determined by gas chromatography to account for the potential transfer of carbon dioxide, methane or sulfide through the membrane. Container storing the gas was flushed with fresh oxygen every 1–3 days. Changes in gas volume present in the container, composition of the gas, and the pressure were used to evaluate the rate of oxygen consumption, using the ideal gas law.

In the case of Reactor 2, oxygen was circulated through the external chamber where the membrane was placed. In this case, oxygen was then transferred towards the lumen of the membrane (outside/in operation for oxygen, see Figure 1b), where liquid from the UASB reactor was flowing (recirculation). A system to manage oxygen gas was set, similar as that described for Reactor 1, including a container for the oxygen, connected to a graduated cylinder containing water. As was the case for Reactor 1, content of the container was flushed every 1–3 days and the same approach was used to evaluate the rate of oxygen consumption. Oxygen was circulated between the mass transfer chamber and the oxygen container at a rate of 40 mL min^-1^. Liquid recirculation of the UASB was set at 45 mL min^−1^.

### 2.2. Reactor Operation

UASB reactors were operated at an organic loading rate (OLR) of 7.5 g-COD L^−1^ d^−1^, and at a sulfur load of 0.075 g-S L^−1^ d^−1^ (COD/S ratio of 100). Diluted wine supplemented with anhydrous sodium sulfate was used as substrate. COD and sulfate concentrations were 7.2 and 0.19 g L^−1^, respectively. UASB reactors were inoculated with anaerobic granular sludge from a full-scale UASB reactor treating brewery wastewater. Both reactors were operated at 35 °C and pH was maintained at 7.2 by the addition of NaHCO_3_ to the feed. During Reactor 1 operation, membrane oxygenation produced no or little effect on sulfide concentration in the biogas. As a result, operation of that reactor was stopped at day 36, as will be discussed below.

The operation included a start-up period (not reported). During the first 10 days of operation (after start-up), no micro-oxygenation was applied to characterize the performance of the system without H_2_S oxidation. On day 11, the described membrane-based micro-oxygenation systems started their operation. During operation, several parameters were determined. They are presented in Table 1, including the analytical methods used. By the end of Reactor 2 operation, the biofilm formed inside the silicone membrane was collected for total solids and sulfur determination. Total solids were determined according to standard methods [18]. Elemental sulfur content of the biofilm was determined by elemental analysis (Isoprime-Euro EA 3000, Eurovector, Pavia, Italy).

### 2.3. Mass Balances

**Figure 2:**
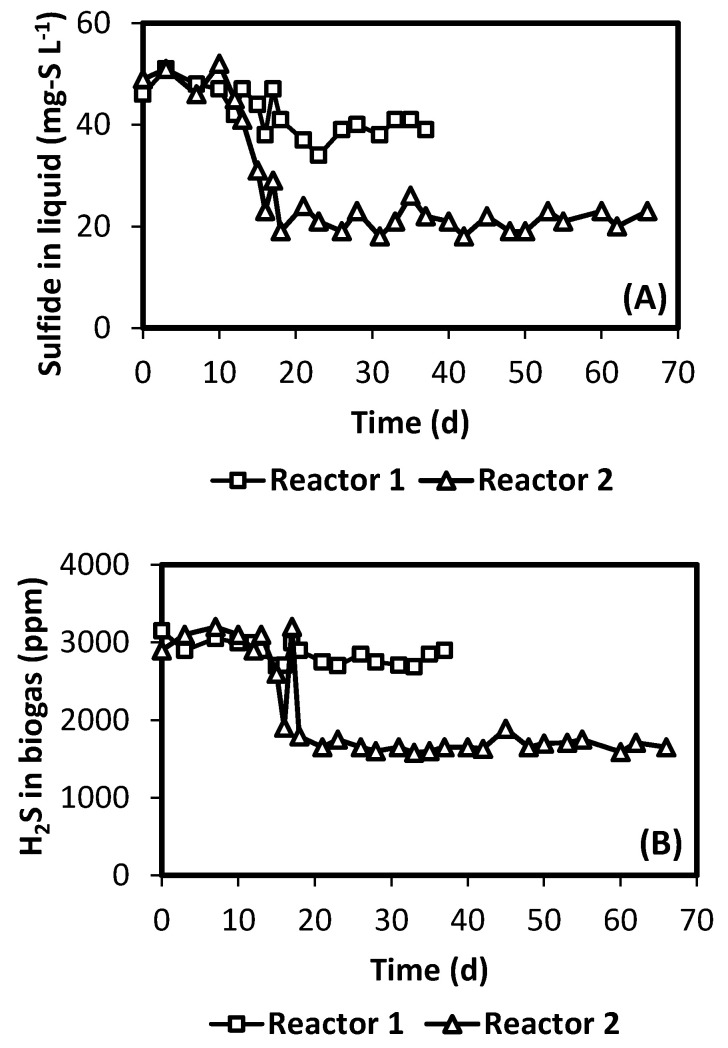
Concentration of sulfide in liquid effluent (**A**) and biogas (**B**) during UASB reactors operation.

As already commented, oxygen consumption was determined by recording the changes in the volume of gas contained in the oxygen container (Figure 2). However, during operation, carbon dioxide, methane, and sulfide were detected in the oxygen container of both reactors, because of back-transport of those species from the liquid phase of the reactor. Then, in order to properly determine the oxygen consumption, changes in total gas volume as well as changes in composition of the gas were considered by means of a mass balance.

Sulfur mass balances were evaluated during Reactor 2 operation. The sulfur entering the system was determined considering the one present in the liquid influent (i.e., sulfate). The sulfur leaving the system was determined considering the sulfur contained in the biogas as H_2_S, the one contained in the liquid effluent as sulfide, sulfate, and sulfite, the one present in the biofilm developed inside the membrane, and the one leaving the system through the membrane in the form of gaseous sulfide. Mass balances were evaluated for the period without oxygenation (days 1–10) and for the period with membrane assisted oxygenation. In the second case, data from day 16 until the end of the operation were considered. Mass balances were calculated considering the whole mass of sulfur species entering and leaving the system, during the periods of time considered.

## 3. Results

### 3.1. Reactor Performance

Both reactors presented similar COD removals during the whole operation period (no oxygenation and oxygenation stages). During the first 10 days of operation (i.e., no oxygenation), the average removal was 87.9% (s = 0.5%) and 91.3% (s = 0.5%) for Reactors 1 and 2, respectively. From day 11 onwards, values were 88.1% (s = 0.3%) and 93.9% (s = 1.2%), respectively. A t-student test confirmed no statistical difference between values with and without membrane micro-oxygenation for Reactor 1. However, in the case of Reactor 2 t-Student test showed that the increase in COD removal was statistically significant (α = 0.05). In addition, no changes were observed in the volumetric biogas production of both reactors, which remained around 6 L d^−1^ (the volume in standard conditions).

Figure 2 presents the contents of sulfide in liquid effluent and biogas, during the operation of both reactors. As shown in Figure 2A, Reactor 1 presented a small decrease in the sulfide content of the liquid effluent after membrane oxygenation was started, from 50 to 40 mg-S L^-1^ (i.e., after day 10). In the case of Reactor 2, sulfide in the liquid effluent decreased from 50 to 20 mg-S L^-1^ in the same period. This decrease in sulfide concentration is most likely related to the increase in COD removal already commented. Moreover, as expected, the decrease in H_2_S concentration levels in the liquid phase of both reactors was related to the decrease in biogas levels. For example, H_2_S in the biogas for Reactor 2 decreased from about 3100 to 1650 ppm (Figure 2B).

The results presented in Figure 2 suggest that membrane oxygenation in Reactor 1 was somehow ineffective, considering the small reduction in sulfide concentration. Low membrane area (half of that in Reactor 2) may have contributed. Moreover, limited mass transfer on the external surface of the membrane may have influenced the response, since the membrane was submerged in the sludge bed, where mixing is limited. In the case of Reactor 2, phases were constantly in circulation, most likely providing better conditions for oxygen transfer and sulfide conversion. As a result of an ineffective sulfide oxidation, the operation of Reactor 1 was stopped at day 36, and only the operation of Reactor 2 continued. As the operation of Reactor 2 advanced, formation of a biofilm was observed in the internal surface of the silicone membrane, which developed as the reactor operation progressed. This was not the case for Reactor 1, which presented no biofilm development on the membrane surface. Development of a biofilm with sulfide oxidation activity, associated with an oxygen transferring membrane, has been indeed reported as an interesting system for successful sulfide oxidation [19,20,21]. Eventually, the biofilm that developed on Reactor 2 clogged the lumen of the membrane, blocking liquid circulation. For this reason, operation of that reactor was stopped at that moment (day 66).

Table 2 presents the amounts of sulfide leaving the system during Reactor 2 operation. Data indicate that micro-oxygenation promoted decreases in sulfide loads associated with UASB liquid effluent and biogas. Overall, sulfide reduction was close to 55%. Most of the sulfide left the system in the liquid phase.

**Figure 3:**
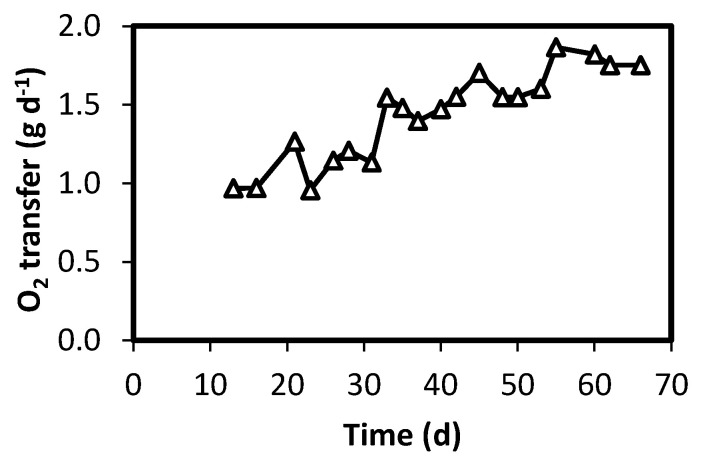
Oxygen mass transfer through the oxygenation membrane in the Reactor 2.

Figure 3 shows O_2_ transfer for Reactor 2, which varied between 1 and 1.8 g-O_2_ d^-1^ (0.45–0.81 g-O_2_ L^-1^ d^-1^). Considering the sulfide produced by Reactor 2 during the first 10 days of operation (Table 2), it can be estimated that the oxygen supply was in the range of 6–11 mol O_2_ per mol sulfide, depending on the operation day. The latter ratio is much higher than that stoichiometrically required for complete sulfide oxidation to sulfate (2 mol O_2_/mol sulfide). Table 2 shows that micro-oxygenation promoted a conversion of 0.0387 g-S L^-1^ d^-1^. Therefore, it can be determined that conversion per unit of membrane area was 2.4 g-S m^2^ d^-1^, based on internal membrane area. This value is in the same range of that reported by Pokorna-Krayzelova et al. [20] when operating a silicone-based biomembrane system for sulfide oxidation. Sahinkaya et al. [22] reported much higher levels of sulfide oxidation (close to 50 g-S m^2^ d^-1^) on a membrane biofilm reactor for treating sulfide-containing effluent. Differences are most likely the result of a different membrane types, and the use of pressurized oxygen to enhance its transfer.

**Figure 4:**
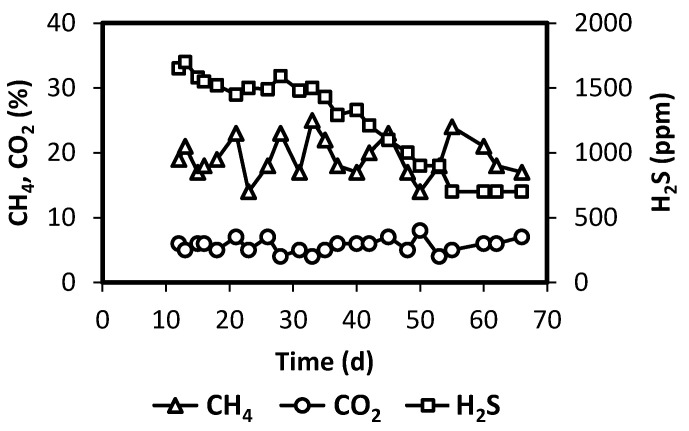
Concentration of sulfide in oxygen container for the Reactor 2 system.

During the operation of Reactor 2, CO_2_, CH_4_, and H_2_S were detected in the oxygen container (element 5 in Figure 1). This was the result of the transport of those compounds through the membrane, from the liquid phase to the gas phase. As already commented, the container storing oxygen was flushed every 1–3 days to prevent excessive oxygen dilution. Concentrations of CO_2_, CH_4_, and H_2_S were determined before flushing the container with fresh oxygen. These are presented in Figure 4. Values indicate that transfer of CO_2_ and CH_4_ were relevant, by the end of operation reaching ranges of 4–7% and 15–25%, respectively. H_2_S was also detected, starting with values close to 1700 ppm, which decreased to 700 ppm by the end of the operation period. It is worthy to notice that the results shown in Figure 4 indicate that the silicone membrane is not selective for oxygen. In fact, Pokorna-Krayzelova et al. [20] showed that permeabilities of H_2_S, CO_2_, and CH_4_ were higher than that of O_2_, in silicon rubber membranes. The mass balance computed for CH_4_ showed that the amount of methane transferred was relevant: between 0.3 and 0.5 L per day (volume in standard conditions). This represents that on average about 9% of all the methane produced left the system through the oxygen container. Research carried out by Pokorna-Krayzelova et al. [23] determined methane losses of 3.7% when operating a silicone biomembrane system for sulfide oxidation.

### 3.2. Sulfur Balance

**Figure 5:**
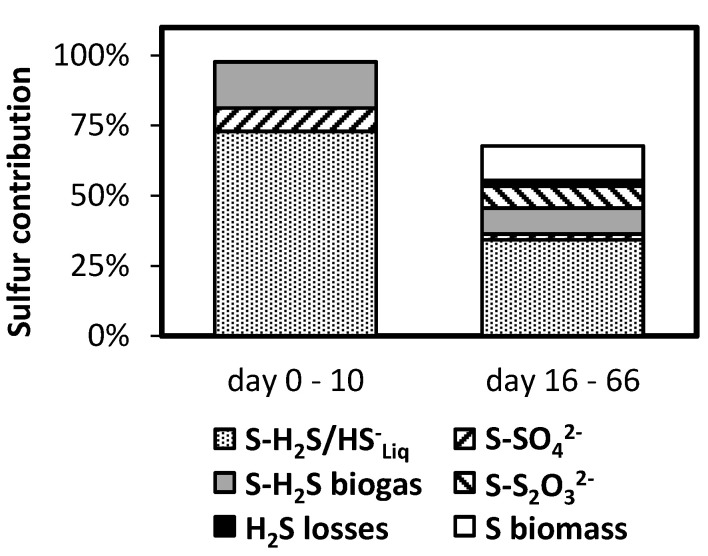
Cumulative contribution of different sulfur compounds to the sulfur leaving Reactor 2 during the indicated periods. Values reported as a percentage of the mass of sulfur that entered the system (as sulfate in the feed).

Figure 5 presents the contribution of the different sulfur species to the total sulfur exiting Reactor 2. The calculation was performed for the operation without and with oxygenation, i.e., days 1–10 and 16–66, respectively. The sum of the total mass of sulfur species leaving the reactor during the studied periods was evaluated. The species considered were sulfide in the biogas, sulfide in reactor effluent, sulfate in the reactor effluent, sulfite in the reactor effluent, sulfur contained in the biofilm formed inside the silicone membrane, and sulfur lost through the membrane. These values are presented as a percentage of the sulfate load applied to Reactor 2 (0.165 g-S d^−1^ or 0.00526 mol-S d^−1^). Then, a value of 100% in Figure 5 indicates that the sulfur that left the system during the considered operation period matches the sulfur that entered the system.

Before oxygenation started (days 1–10), the measured sulfur species accounted for almost 100% of the applied sulfur load. The difference between entering and exiting sulfur was only 2%, a result that supports the procedure used for mass balance determination. During the nonoxygenated period, most of the sulfur left the system dissolved in the liquid phase (about 73%). Sulfide content of the biogas accounted for 17%. Distribution of dissolved sulfide species (HS^-^ and H_2_S) is a strong function of pH, considering that pK_a_ is close to 7. Moreover, this distribution will also affect sulfide equilibrium between liquid and gas phases, by determining the concentration of H_2_S, the volatile sulfur form [24].

Figure 5 shows a decrease in the sulfide leaving the system, both in the biogas and in the liquid phase, when micro-oxygenation was applied. This is the result of the reduction in sulfide concentration in those phases (Figure 2, Table 2). The sulfur leaving the system due to transport through the membrane was also considered (H_2_S losses in Figure 5). It accounted for 2% of the total sulfur leaving the system. Sulfite and sulfate together contributed with 10% of the sulfur leaving the system. Sulfur present in the biofilm formed in the membrane lumen was also determined, representing slightly over 12%. Figure 5 shows a gap of close to 30% in the sulfur mass balance when oxygenation was applied. This means that a large fraction of the incoming sulfur was not identified in the sulfur species tested. During the operation of a microaerated UASB reactor, Krayzelova et al. [1] observed that 33% of the applied sulfur left the system as elemental sulfur, suspended in the reactor effluent. During this research, elemental sulfur was not determined in the liquid effluent of the UASB, which may explain the sulfur gap observed in Figure 5.

## 4. Discussion

Microaeration has proven to be a simple, reliable, and inexpensive way to control the sulfide content of the biogas and the liquid effluent. Moreover, several studies have reported that small doses of oxygen may enhance anaerobic digestion by improving hydrolysis and/or acidification [14,25]. However, depending on the reactor configuration, ensuring an efficient provision of oxygen may not be a simple task. Most of reported research and applications have been focused on microaeration of continuous stirred-tank reactors, where air/oxygen is normally injected into the headspace of the digester, and less research has been dedicated to the application of microaeration in granular reactors [14]. The development of successful strategies to apply microaeration in granular reactors may extend the benefits of sulfide removal by in situ oxidation to the anaerobic treatment of sulphate-rich wastewaters. Gas permeable membranes may be a way to achieve such a goal.

The configuration in which the membrane was placed in the sludge bed (Reactor 1) failed to provide a relevant decrease in sulfide concentration. A submerged membrane configuration may not then be a suitable alternative for an oxygenation system, considering the observed performance. Moreover, access to the interior of a full-scale reactor for installation or maintenance of such a system would not be practical. In an external membrane module, operation flow of phases can be better controlled, mass transport can be then enhanced, and easy access to the system is ensured.

Only few reports are available dealing with microaeration of UASB reactors. Zhou et al. [26] reported 20–30% of H_2_S removal in a UASB treating evaporator condensate from a sulfite pulp mill. On the other hand, Krayzelova et al. [1] achieved 73% sulfide removal, when treating a synthetic wastewater. In both cases, UASB reactors were microaerated by injecting air directly into the reactor. In this research, Reactor 2 provided 55% sulfide removal. These values of sulfide removal are lower than those reported for mixed reactors with air injection in the headspace. Nevertheless, these results may be considered as promising and could lead to the successful implementation of micro-oxygenation of granular reactors for wastewater treatment. On the other hand, membrane aeration could represent an effective way to provide oxygen in a controlled way and may provide conditions for the development of sulfide oxidation microorganisms, as observed by Camiloti et al., [19] when operating an equivalent setup.

During Reactor 2 operation, oxygen consumption was largely higher than the stoichiometric requirement. Therefore, it is inferred that O_2_ permeability of the silicone membrane did not limit the efficiency of sulfide removal. Excess O_2_ may have been used in the biofilm for substrate aerobic oxidation. Another potential route of oxygen consumption could be the establishment of a cycle of oxidation/reduction of sulfur in the membrane module/reactor system. Sulfate reducing bacteria can use sulfate, thiosulfate, or even sulfite as an electron acceptor in the process of dissimilatory sulfate reduction by SRB [27,28,29]. Therefore, there is a chance that the production of oxidized sulfur products, generated by SOB, triggered a new reduction process, causing a higher consumption of oxygen. Indeed, sulfate and sulfite concentrations in the liquid effluent of the UASB reactor increased during the operation period when membrane oxygenation took place. Control of the oxygen dose to prevent under- or over-oxygenation is indeed a challenge that may be addressed by precise automatic process control [25]. Relevant levels of methane losses were identified during system operation. This may seriously jeopardize the sustainability of membrane assisted oxygenation systems for sulfide control. Methane losses are a result of mass transport through the membrane, since reactor concentration in the liquid phase is normally close to saturation. Therefore, the development and/or selection of membrane materials are decisive to promote a higher oxygen transfer rate for oxygen, and a lower one for methane. Moreover, detailed determination of the required membrane area is key to provide conditions for the transfer of only the required oxygen, limiting excessive methane losses.

During this research, a tubular membrane was used, i.e., a single 2.2 m length and 5 mm internal diameter tube. Biomass growth within the tube caused membrane clogging before 2 months of operation. A configuration ensuring an easy access to the membrane may be more adequate, facilitating the removal of excess biomass that develops as biofilm. The modification of commercially available membrane modules may be a simple and affordable way to implement a micro-oxygenation module for sulfide oxidation. For example, Pokorna-Krayzelova et al. [23] used a commercially available microfiltration membrane for biogas desulfurization on a pilot scale, with positive results.

## 5. Conclusions

The use of a membrane-based oxygenation system is an interesting alternative to provide conditions for sulfide oxidation, reducing the concentration of this compound both in the liquid effluent as well as in the biogas, in granular-based anaerobic reactors like UASB. The use of an external membrane module, connected to the reactor by the recirculation line, seems to be a convenient way to do so, since it would facilitate access and maintenance. In this research, a membrane that was nonselective for sulfide was used, resulting in methane loses (about 9%). Membrane selection and operation to reduce methane loses is required to ensure the sustainability of membrane-oxygenation of UASB reactors. Even though partial sulfide oxidation was observed in this research (55% removal), the results are considered promising, since they may lead to the successful implementation of micro-oxygenation of granular reactors for wastewater treatment.

## Figures and Tables

**Table 1:** Parameters determined during system operation.

Parameter	Method	Periodicity
Chemical oxygen demand (COD)	Standard methods [18]	2–3 times/week
Biogas volumetric production	Water displacement	2–3 times/week
Gas and biogas composition (CH_4_/CO_2_/N_2_/O_2_)	Gas chromatography with TCD detector (Clarus 500, Perkin Elmer, Waltham, MA, USA)	2–3 times/week
Sulfate	Ionic chromatography (Compact IC plus 882, Methrom, Herisau, Switzerland)	2–3 times/week
Thiosulfate	Ionic chromatography (Compact IC plus 882, Methrom, Herisau, Switzerland)	2–3 times/week
Dissolved sulfide	Spectrophotometry (sulfide reagent set, methylene blue, product number 2244500, Hach, Loveland, CO, USA)	2–3 times/week
Biogas sulfide	Gas chromatography with FPD detector (Clarus 500, Perkin Elmer, Waltham, MA, USA)	2–3 times/week

**Table 2:** Sulfide loads leaving the system during Reactor 2 operation. Averages over the indicated periods are presented.

Parameter	Units	Days 1–10 (No Micro-Oxygenation)	Days 16–66 (with Micro-Oxygenation)
Sulfide leaving the system in the gas phase	(g-S L^−1^ d^−1^)	0.0122	0.0067
Sulfide leaving the system in the liquid phase	(g-S L^−1^ d^−1^)	0.0585	0.0253
Sulfide leaving the system (Total)	(g-S L^−1^ d^−1^)	0.0707	0.032
